# Frequency of personal care product use among reproductive-aged Black individuals and associations with socio-demographic characteristics

**DOI:** 10.1038/s41370-024-00690-x

**Published:** 2024-05-29

**Authors:** Kyla W. Taylor, Caroll A. Co, Symielle A. Gaston, Chandra L. Jackson, Quaker Harmon, Donna D. Baird

**Affiliations:** 1 Division of Translational Toxicology, National Institute of Environmental Health Sciences, National Institutes of Health, Department of Health and Human Services, Research Triangle Park, NC USA; 2 Social and Scientific Systems, Inc., a DLH Holdings Corp Company, Durham, NC USA; 3 Epidemiology Branch, National Institute of Environmental Health Sciences, National Institutes of Health, Department of Health and Human Services, Research Triangle Park, NC USA; 4 Division of Intramural Research, National Institute on Minority Health and Health Disparities, National Institutes of Health, Department of Health and Human Services, Bethesda, MD USA

**Keywords:** Chemicals in Products, Environmental Justice, Epidemiology, Personal Exposure, Vulnerable Populations

## Abstract

**Background:**

Compared to White women, Black women in the United States are more likely to use personal care products (PCPs) with higher concentrations of endocrine-disrupting chemicals (EDCs) and harsher chemical formulations. This may contribute to differential health outcomes in Black women such as increased risk of breast cancer, cardiometabolic outcomes, adverse birth outcomes, and uterine fibroids.

**Objective:**

Classify distinct PCP use patterns across multiple types of products and examine how patterns vary by socio-demographic characteristics.

**Methods:**

The Study of Environment, Lifestyle and Fibroids is a cohort study of reproductive-aged Black individuals living around Detroit, Michigan. Using self-reported data on frequency of PCP collected between 2013–2018, we employed latent class analysis to identify distinct groups of participants with similar PCP use. Socio-demographic characteristics were compared across latent classes.

**Results:**

Among 1562 participants, we identified 6 latent classes: Lower Overall; Higher Nailcare; Higher Skincare; Moderate Overall; Higher Makeup/Haircare/Skincare; Higher Overall. Makeup and nailcare usage were the most predictive for classifying participants into groups. Participants in classes with less frequent use of all PCPs and those with only high use of nailcare products, were more likely to report lower socio-economic status (SES), be current smokers, have a body mass index of ≥35 kg/m^2^, and have ≥3 births. In comparison, participants in classes with average and more frequent use of PCPs were more likely to report higher SES, be non-smokers, be nulliparous, and have ever used oral contraceptives.

**Impact statement:**

This study is one of the first detailed assessments of PCP usage among a large cohort of young adult Black women that considers multiple product categories including makeup, hair, skin, nail, and vaginal products. Latent class analysis was used to capture complex patterns of PCP use and identify distinct groups of individuals with similar product use. Although the latent classes are specific to this study population, the identified socio-demographic characteristics or behaviors associated with latent classes may inform targeted and impactful exposure reduction strategies in similar populations.

## Introduction

Compared to White women, Black women in the United States are exposed to higher and more hazardous concentrations of endocrine-disrupting chemicals (EDCs), which are found in personal care products (PCPs) [1–6]. PCP use differs by race/ethnicity across multiple product categories such as hair products, skin products, and vaginal products [1, 3, 7**–**9]. The use of products in these categories among Black women is rooted in a long history of institutionalized racism that idealizes European beauty standards of straighter hair and lighter skin and pushes unnecessary use of deodorizing and douching products based on unfounded notions of vaginal odors [9, 10]. Targeted marketing to Black women continues to reinforce these historical cultural norms [9]. Products specifically advertised to Black women have been found to have higher concentrations of some EDCs (e.g., phthalates) and more harmful chemical formulations [3, 4, 9]. Therefore, the use and more frequent use of PCPs likely increases Black women’s risk of hormone-sensitive health outcomes (e.g., increased risk of breast cancer, cardiometabolic outcomes, adverse birth outcomes, and uterine fibroids).

Several studies have reported associations between hair products commonly used by Black women and their health effects. For example, the use of hair oil and hair relaxers/straighteners has been associated with earlier age of menarche [11, 12], increased risk of breast and uterine cancer [13, 14], and higher incidence of uterine fibroids [15]. In the Study of Environment, Lifestyle, and Fibroids (SELF), a cohort of reproductive-aged Black individuals with an intact uterus at enrollment, we previously used latent class analysis (LCA) to investigate concurrent usage patterns of multiple hair products [16]. Participants who reported more frequent use of hair products (e.g., moisturizers and conditioners) also reported higher socio-economic status (SES). However, because this study was limited to hair products, it did not capture concurrent product use across multiple types of products that occur in real-world settings. Other studies that have examined differences in product use by SES did not have large enough sample sizes to examine these differences among Black individuals [17, 18].

To address this gap in the literature on socio-economic differences in PCP use among Black women, and to expand on previous work [16], we examined the frequency of concurrent product use across a wider range of PCPs and their association with socio-demographic characteristics.

## Methods

### Study Population

We analyzed cross-sectional data of the Study of Environment, Lifestyle, and Fibroids (SELF), a prospective cohort study of reproductive-aged Black individuals living in the Detroit, Michigan area. Further description of the SELF-study design, recruitment and enrollment protocol, and participant characteristics is previously described in Baird et al [19]. Briefly, between 2010–2012, individuals living in the Detroit, Michigan area were eligible for enrollment if they reported that they self-identified as African American and/or Black, were 23–35 years of age, had an intact uterus (which was confirmed by vaginal ultrasound at a clinic visit), and no prior clinical diagnosis of uterine fibroids. To determine eligibility, potential participants answered “yes” or “no” to whether they identified with each of the following racial/ethnic categories: American Indian or Alaska Native, Asian, Native Hawaiian or Other Pacific Islander, Black or African American, White, and separately Hispanic or Latina. Potential participants were considered eligible if they chose any combination of race or ethnicity categories that included Black or African American. Eligible participants (*n* = 1693) completed computer-assisted telephone interviews (CATI), computer-assisted web interviews (CAWI), and clinical examinations at baseline. We did not query participants about their gender, so participants may vary in their gender identities. During the CATI and CAWI interviews, participants self-reported current socio-demographic and lifestyle/health behavior characteristics. Participants were prospectively followed for 5 years, with three follow-up clinic visits at approximately 20-month intervals with similar study activities. The current analysis included participants who filled out the long version of the Household and Personal Care Products module in the CAWI questionnaire during the second follow-up visit in 2014–2016 (*N* = 1445) or during the third follow-up visit in 2016–2018 (*N* = 127) if the second follow-up visit had been missed. Participants who did not have a clinic visit at either second or third follow-up were excluded from analysis (*N* = 121). Participants included and excluded from this study had comparable baseline socio-economic characteristics (Table S1). Participants with missing values on any of the products included in the latent class model were also excluded from the analysis (*N* = 10). The final sample size used in the analysis was 1562 individuals; all of whom provided written informed consent. The SELF protocol was approved by the National Institute of Environmental Health Sciences and the Henry Ford Health Institutional Review Boards.

### Personal care product use

Detailed self-reported use of 48 PCPs over the previous 12 months was collected with questions about frequency of use. Response options for the frequency of use varied between 6 to 7 categories and differed by product. For example, frequency of use response options for eye make, foundation, blush, and bronzer were ≥2 times/day, 1 time/day, 2–6 times/week, 1 time/week, 1–3 times/month, <1 time/month, or did not use and response options for lipstick and lip balm were ≥6 times/day, 2–5 times/day, 1 time/day, 2–6 times/week, 1 time/week, 1–3 times/month, or rarely/never. The frequency of use response options for each product can be found in Table S2. To facilitate the model fitting process, response categories for each product were collapsed into 2 to 4-category levels (always, often, sometimes, and never) from the original 6 to 7-category levels collected in the questionnaire (see Table S3), depending on the distribution of the counts. The responses on the use of hair moisturizing (petroleum jelly, shea butter, natural plant-based oils, hair food, moisturizing creams and lotions, and conditioners), and hair coloring (henna, rinses, semi- and permanent hair dyes, hair bleach) PCPs were combined. The use of perfume, cologne, and body spray or mist were combined to reflect the use of fragrance. The use of antibacterial products contained many “Don’t know” responses (42%) and most of the cohort (98%) did not report the use of growth solution for eyelashes. Therefore, these variables were not included in the analysis. Analyses were performed on 37 PCPs (Table S3).

### Socio-demographic, lifestyle, and reproductive characteristics

Data on socio-demographic, lifestyle, and reproductive characteristics were taken from the corresponding follow-up visit (second or third) when the participants filled out the long version of the Household and Personal Care Products module. Socio-demographic correlates included age at clinic visit (<30 years, 30 to <33 years, 33 to <36 years, and 36 years and over), marital status (never married, previously married, or lived with someone as married, currently married or living with someone as married), educational attainment at time of visit (≤ high school/GED, some college/associates/technical degree, ≥Bachelor’s degree or higher), highest educational attainment of participant’s primary caregiver at age 10 years (≤ high school/GED, some college/associates/technical degree, ≥Bachelor’s degree or higher), total annual household income (<20 K, 20–50 K, 50 K+), and current employment (not employed, employed <30 h per week, employed 30 h or more per week). Lifestyle, including behavioral factors, including measured body mass index (BMI) (<25 kg/m^2^, 25–30 kg/m^2^, 30-< 35 kg/m^2^, ≥35 kg/m^2^), physical activity^1^ (low, low to moderate, high, very high), smoking status (non-smoker, former smoker, <10 cigarettes per day, 10 or more cigarettes per day), and recent and highest alcohol use (low: <10 drinks/year, moderate: up to 5 drinks on days when having alcohol or no more than a single occasion per month with 4+ drinks, heavy: 6 or more drinks on days when having alcohol or 4+ drinks on 2 or more occasions per month). Reproductive factors included age at menarche in years (<11, 11, 12, 13, ≥14), parity (nulliparous, 1, 2, 3 or more births), ever used oral contraceptives (no, yes), ever used depo (no, yes), use of lubricated condoms or spermicides in past 12 months (did not use, used), and contraception at visit [none, birth control pills with both estrogen and progestin, progestin-only pills, hormonal implant, Depo-Provera, hormonal IUD (intrauterine device) or Mirena, non-hormonal IUD, vaginal ring, patch]. The categories of participant characteristics used in this analysis are consistent with our previous analysis [16]. These categories generally reflect the categories offered on the questionnaire which were chosen to reflect meaningful differences and reduce participant burden. Some categories are collapsed when there were small numbers (e.g., household income also included a category for >$100k but fewer than 3% of participants selected that category so it was combined with >$50 K.

### Behaviors related to personal care product ingredients

We also looked at questions relating to participants’ behaviors within the past 12 months on the choice of PCP based on product ingredients. Separate questions were asked frequency of use of each of the following fragrance-free products: soap or body wash, skin creams, deodorant/anti-perspirant, and panty liners/pads/tampons. Response options were as follows: frequently or always, occasionally, rarely or never. Separate questions were asked about whether participants avoided products with (1) parabens, (2) bisphenol A, or (3) triclosan with response options of no or yes.

### Statistical Analysis

We used latent class analysis (LCA), a model-based unsupervised clustering approach used for detecting and discovering group structure in data with multivariate categorical responses [20–22]. Due to the large number of products considered in the analysis, a variable selection technique was utilized to facilitate model fitting and to simplify model interpretation. We used the variable selection technique proposed by Dean & Raftery et al. [23] and implemented it in the LCAvarsel package (v1.1) in R (v4.1.2). To ensure that the final model had representation from each main product group, we employed a two-step process where variable selection was first performed within each product group as a pre-screening step. An exception to the pre-screening step was made for vaginal products since this group only had 3 products belonging to this category. Variable selection was performed in two rounds. Variables selected in the pre-screening step were chosen as the starting set in the second round of variable selection. Products not included in the starting set are still evaluated in the algorithm. We allowed the algorithm to fit models ranging from 2 to 8 latent classes. The algorithm selects the set of variables most predictive of clustering and the optimal number of latent classes by choosing the model with the smallest Bayesian Information Criterion (BIC). Furthermore, we added a constraint for all latent classes to contain at least 10% of participants, to ensure stability of estimates in subsequent analyses. We used PROC LCA (v1.3.2) in SAS (v9.4) to fit the final model. We initialized the model with 100 random starting values and chose the starting value that produced the lowest BIC as the final solution.

The resulting posterior probabilities from the LCA model were used to classify subjects into latent classes. We assigned subjects to the latent class in which they had the highest probability of membership. To understand which products were driving the results of the LCA model, we conducted chi-squared tests of association between the assigned class membership and each product included in the model. We then compared the log worth (-log_10_(*p*-value)) across all products to ascertain the relative importance of each product to the latent classes. All analyses were performed in R (v4.1.2) and SAS (v9.4).

Associations between latent class membership and socio-demographic, reproductive, and lifestyle factors were analyzed using chi-squared tests.

## Results

### Study population

The average age in this cohort at the time of the second or third visit was 32.6 years with a standard deviation of 3.4 years. Among the entire cohort, 41% were currently married, 33% had a bachelor’s degree or higher, 36% had an annual household income of <20k, and 22% were not currently employed. Seventy percent of participants were non-smokers, and the majority (67%) reported moderate alcohol consumption. About half of the participants (52%) reported low to moderate or moderate physical activity and 45% had a BMI > 35 kg/m^2^. Most participants experienced first menses between ages 11–13 years (64%), had at least one live birth (68%), and reported ever use of oral contraceptives (73%) (Table 1).

**Table 1:** Distribution of self-reported socio-demographic, lifestyle, and reproductive characteristics of participants overall and in each latent class of personal care product usage, SELF (2013–2018), *N* = 1562.

Characteristics		Classes of personal care products use							
		All Participants	Class 1 Lower Overall	Class 2 Higher Nailcare	Class 3 Higher Skincare	Class 4 Moderate Overall	Class 5 Higher Makeup/ Haircare/ Skincare	Class 6 Higher Overall	*p*-value
	*N* (%)	1562	279 (18)	192 (12)	257 (16)	386 (25)	238 (16)	210 (13)	
Age	<30 years	26	29	28	22	28	21	28	0.25
	30 to <33 years	26	22	28	24	28	27	25	
	33 to <36 years	27	30	20	30	26	30	26	
	36 years and over	21	20	24	25	19	22	21	
Marital status	Never married	39	42	37	42	37	38	37	0.82
	Previously married or lived as married	20	18	25	19	19	21	20	
	Currently married	41	41	39	40	44	40	43	
Educational attainment at F2/F3	High school/GED or less	16	28	29	18	8	6	11	<0.0001
	Some college/associates/ technical	51	55	57	53	47	42	59	
	Bachelors/Masters/PhD	33	17	15	29	46	53	31	
Childhood: Highest educational attainment of primary caregiver	High school/GED or less	47	63	60	48	35	36	46	<0.0001
	Some college/associates/ technical	42	30	35	44	49	48	42	
	Bachelors/Masters/PhD	12	7	5	9	17	16	12	
Total annual household income at visit	<20K	36	55	51	39	23	23	31	<0.0001
	20–50K	39	33	38	36	42	40	45	
	50K+	25	12	12	25	35	37	24	
Current employment at visit	Not employed	22	36	29	21	16	16	19	<0.0001
	Employed <30 hours/week	9	10	12	9	10	7	10	
	Employed 30+ hours/week	68	54	59	70	74	78	71	
Body Mass Index	Less than 25 kg/m^2^	16	17	12	16	17	18	15	0.040
	25–30 kg/m^2^	19	16	16	17	20	25	21	
	30 to <35 kg/m^2^	20	17	19	21	20	21	22	
	35 kg/m^2^ and above	45	51	54	47	43	35	41	
Smoking	Non	70	53	59	72	79	81	68	<0.0001
	Former	9	15	5	11	8	6	10	
	Current (<10 cigarette/day)	16	22	29	13	9	11	20	
	Current (10+ cigarette/day)	5	10	7	5	4	2	2	
Alcohol use^a^	None	14	23	14	22	11	8	8	<0.0001
	Moderate	67	58	63	63	73	71	70	
	Heavy	19	20	23	15	16	21	22	
Alcohol use at heaviest	None	7	12	6	10	6	4	6	0.0001
	Moderate	44	41	39	46	49	51	36	
	Heavy	48	47	56	44	45	45	58	
Physical activity (at F2^b^)^c^	Low	20	21	20	22	19	19	19	0.23
	Low to moderate	26	25	26	26	23	30	23	
	Moderate	26	31	24	24	30	22	27	
	High	17	11	19	18	14	21	16	
	Very high	11	12	11	11	14	7	15	
Physical activity (at F2^b^)	<150 mins of vigorous activity or <7 hrs of moderate activity/week	72	77	71	72	73	71	69	0.51
	≥150 mins of vigorous activity or >7 hrs of moderate activity/week	28	23	29	28	28	29	31	
Age at menarche	Early menarche	19	22	15	21	18	18	17	0.56
	11 years	20	19	21	20	22	22	16	
	12 years	27	30	23	27	28	22	28	
	13 years	17	14	20	15	15	19	20	
	Late menarche	18	16	21	18	17	18	20	
Parity	Nulliparous	32	23	21	34	38	44	31	<0.0001
	1	25	23	26	25	26	23	25	
	2	20	20	23	19	21	20	21	
	3 or more	23	34	31	22	16	14	23	
Ever used oral contraceptives	No	27	33	35	29	22	22	21	0.0003
	Yes	73	67	65	71	78	78	79	
Ever used depo	Never	55	51	41	55	59	67	51	<0.0001
	Ever	46	50	59	46	42	34	49	
Used lubricated condoms/spermicides in past 12 months	Did not use	62	68	58	70	61	62	52	0.0005
	Used	38	32	42	30	39	38	48	
Most likely form of contraception^d^ at clinic visit	None	73	80	80	73	69	68	70	0.004
	BCP	9	6	5	12	10	11	9	
	Hormonal implant or POP	2	3	1	0	3	1	3	
	Depo-Provera	5	7	4	7	4	5	5	
	Hormonal IUD or Mirena	7	4	7	6	8	7	9	
	Non-hormonal IUD	3	1	3	1	3	5	4	
	Vaginal ring or Patch	1	1	1	0	3	3	1	

Values reported are column percentages, which may not add up to 100 due to rounding. Observations with missing values were not included; *P*-values correspond to Chi-squared test for differences in distribution across the 6 latent classes; *BCP* Birth control pill, *POP* Progestin-only pill, *IUD* Intrauterine device.

^a^Alcohol use: Moderate (1–5 drinks on days when having alcohol or 4+ drinks once/month or less); Heavy (6+ drinks on days when having alcohol or 4+drinks minimum 2x/month).

^b^Only women who had F2 data were included in the analysis of physical activity.

^c^Physical activity: Low (<1 hour/week of vigorous activity, 2 hours/week of moderate activity, and 14 hours/week of walking); Low to moderate (MET score below 72); Moderate (MET score above 72); High (150–300 mins/week of vigorous activity or 7 to 10 hours/week of moderate activity); Very high (≥300 mins/week of vigorous activity or ≥10 hours/week of moderate activity).

^d^For most likely forms of contraception at clinic visit, women who responded with non-contracepting progesterone (*n* = 5), unknown IUD (*n* = 1), and other fertility medication (*n* = 1) were excluded from the analysis. Infrequently reported forms of hormonal contraception were grouped according to the hormone composition; progestins only, estrogen, and progestins.

### Latent classes of personal care product use

A table with the products considered in the modeling process is provided in Tables S2, S3. The variable selection process resulted in a model containing 5 makeup products (foundation, blush, bronzer, lipstick, and eye makeup); 4 skin products (face/hand/body creams, perfume, or body spray); 4 nail products (gel polish, gel nail extension, gel overlays, acrylic overlays); 3 hair products (hair creams or conditioners, hair styling products, hair dyes); and 3 vaginal products (douche, talc, vaginal lubricant). Details of the variable selection process are shown in Table S4. Seven and eight latent classes produced the lowest BIC and AIC, respectively (Table S5). However, both models contained at least one latent class with less than 10% of all observations, so we chose to use the 6-class solution (the next best solution) instead. An examination of latent class posterior probabilities showed that over 92% of participants had a probability of at least 0.60 in the class they were assigned to, 80% had probabilities over 0.80 and 69% had probabilities over 0.90, suggesting participants were well differentiated across classes. The distribution of posterior probabilities by latent class is shown in Figure S1.

Latent classes identified in the LCA model showed different patterns in the frequency and types of products used within the SELF cohort. The proportion of participants assigned to each class ranged from 12% to 25%. Latent class labels were assigned to the six latent classes based on comparing probabilities of usage frequency categories in each latent class to probabilities of usage frequency categories in the overall study population. Latent classes were reordered from lowest frequency of use to highest frequency use to facilitate interpretation and visualization of results. The latent class names, proportions, and descriptions are as follows: (1) Lower Overall (18%): lower/less frequent use of products across all categories; (2) Higher Nailcare (12%): lower/less frequent use of most products but higher/more frequent use of nail products; (3) Higher Skincare (16%): lower/less frequent use of most products but higher/more frequent use of body and hand creams; (4) Moderate Overall (25%): class closest to the cohort average; (5) Higher Make-up/Haircare/Skincare (16%): higher/more frequent use of makeup, skincare, and haircare products but lower/less frequent use of nailcare products; (6) Higher Overall (13%): similar to the Higher Makeup/Haircare/Skincare class but with additional higher/more frequent use of nailcare products.

**Fig. 1:**
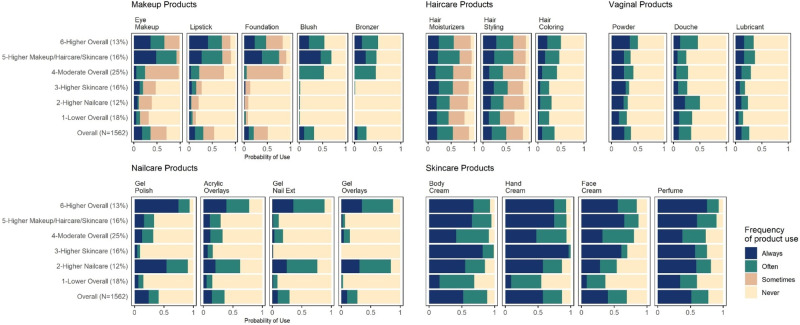
Item-response probabilities estimated from a latent class analysis model with 19 personal care products and 6 latent classes, SELF (2013–2018), *N* = 1562. The frequency distribution of each product for the entire cohort is shown in the last row (Overall). Latent class labels were assigned based on comparing probabilities of usage frequency categories in each latent class to probabilities of usage frequency categories in the overall sample. Latent classes: (1) Lower Overall (18%): lower/less frequent use of products across all categories; (2) Higher Nailcare (12%): lower/less frequent use of most products but higher/more frequent use of nail products; (3) Higher Skincare (16%): lower/less frequent use of most products but higher/more frequent use of body and hand creams; (4) Moderate Overall (25%): class closest to the cohort average; (5) Higher Makeup/Haircare/Skincare (16%): higher/more frequent use of makeup, skincare, and haircare products but lower/less frequent use of nailcare products; (6) Higher Overall (13%): similar to the Higher Makeup/Haircare/Skincare class but with additional higher/more frequent use of nailcare products.

The parameter estimates, or the item-response probabilities estimated from the model, shown in Fig. 1, represent the probabilities of PCP use conditional on the latent class membership. The results from the analysis comparing the log worth (-log_10_(*p*-value)) across products showed that products in the makeup and nailcare categories constituted the highest log worth, indicating that these products were the main drivers of the clustering results. Haircare and vaginal products, although they still had some contribution to the model, were not as significant relative to other product groups. Figure S2.

### Associations between latent class and socio-demographic, lifestyle, and reproductive factors

Distribution of correlates within each latent class and the corresponding results from conducting Chi-squared tests for all factors are displayed in Table 1. Age distribution across latent classes were similar (*p* = 0.25). Educational attainment, annual household income, highest educational attainment of primary caregiver at childhood, and current employment had more notable differences between classes (*p* < 0.001). When compared to the other four classes, membership in the Lower Overall and Higher Nailcare classes, which represent lower/less frequent PCP use, except for higher/more frequent use of nailcare products in the latter, was associated with lower SES (17% and 15% attained a bachelor’s degree or higher; 36% and 29% were unemployed; >50% in both classes had annual household incomes of <20k). When compared to the Lower Overall and Higher Nailcare classes, the Moderate Overall and Higher Makeup/Haircare/Skincare classes were associated with higher SES (46% and 53% attained a bachelor’s degree or higher, 23% in both classes had annual household incomes <20k, and 16% in both classes were unemployed). Despite having different product use patterns, the Higher Skincare and Higher Overall classes, representing lower/less frequent use of most products except for body and hand creams (Higher Skincare class) and more frequent use of makeup, haircare, nailcare, and skincare products (Higher Overall class), had similar SES (29% and 31% attained a bachelor’s degree or higher, 39% and 31% had annual household incomes <20k, 21% and 19% were unemployed).

A similar pattern was found with smoking where the Lower Overall and Higher Nailcare classes had the highest percentage of current smokers (32% and 36%) (*p* = <0.0001). In contrast, the Moderate Overall and Higher Makeup/Haircare/Skincare classes had the highest percentage of non-smokers (79% and 81%, respectively). For alcohol use, the Lower Overall and Higher Skincare classes tended to have more non-drinkers (23% and 22%) relative to other classes (*p* = <0.0001). For BMI, we found slight differences (*p* = 0.04) in distribution across classes. The Lower Overall, the Higher Skincare, and the Higher Nailcare classes had the highest percentages of participants with a measured BMI of ≥35 kg/m^2^ (51%, 47%, and 54% respectively). In contrast, the Moderate Overall, Higher Makeup/Haircare/Skincare, and Higher Overall classes had the lowest percentage of participants with a measured BMI of ≥35 kg/m^2^ (43%, 35%, and 41%, respectively). The Higher Makeup/Haircare/Skincare class had the highest percentage of participants with a measured BMI of 25–30 kg/m^2^ (25%) relative to the other latent classes. We did not find any association between recreational physical activity and latent classes (*p* = 0.23) in this cohort.

We did not find a significant association between marital status and latent classes (*p* = 0.82); however, parity status was associated with latent classes (*p* = <0.0001). The Moderate Overall and Higher Makeup/Haircare/Skincare classes were more likely to be nulliparous (38% and 44%) and the Lower Overall and Higher Nailcare classes were more likely to have participants with 3 or more births (34% and 31%). Ever use of hormonal contraceptives also varied between classes with participants in the Moderate Overall class, the Higher Makeup/Haircare/Skincare class, and the Higher Overall class having the highest percentage of participants reporting having ever used oral contraceptives (78%); the Higher Nailcare class had the highest percentage (59%) of participants who have ever used Depo Provera; and the Higher Nailcare and Higher Overall classes were more likely to have participants who have used lubricated condoms or spermicides in the past 12 months (42% and 48%).

### Associations between latent classes and behaviors related to personal care product ingredients

We did not find a consistent pattern across latent classes for behaviors relating to use of fragrance-free (FF) products (Table 2). Participants in the Higher Nailcare, Moderate Overall, and Higher Overall classes were more likely to occasionally or frequently use FF skin creams (57%, 55%, and 59% respectively) (*p* = 0.0036). The majority (66%) of the cohort did not use FF deodorant or antiperspirant. Among the latent classes, participants in the Higher Makeup/Haircare/Skincare class had the smallest percentage (9%) of participants frequently using FF deodorant or anti-perspirant. The majority of the population either frequently used (45%) or occasionally used (20%) FF panty-liners, pads, or tampons. Participants in the Higher Nailcare, Moderate Overall, and Higher Makeup/Haircare/Skincare classes reported the highest frequency use of FF vaginal products (47%, 47%, and 51%), while participants in the Higher Overall class reported the highest occasional use of these products (27%) (*p* = 0.0005). No marked differences in preference for FF soap or body wash were observed across latent clusters (p = 0.82). Although most of the cohort did not avoid products with parabens, BPA or triclosan (83%, 81%, and 91%), among the latent classes, the Moderate Overall and Higher Makeup/Haircare/Skincare classes had the highest percentage of participants who avoid products with parabens (20% and 26%; *p* = 0.0003) and BPA (24%; *p* = 0.0010). We did not find a significant difference in triclosan avoidance across latent classes (*p* = 0.31).

**Table 2:** Distribution of behaviors related to personal care product ingredients by latent class, SELF (2013–2018), *N* = 1562.

Characteristics		Classes of personal care products use							
		All Participants	Class 1 Lower Overall	Class 2 Higher Nailcare	Class 3 Higher Skincare	Class 4 Moderate Overall	Class 5 Higher Makeup/ Haircare/ Skincare	Class 6 Higher Overall	*p*-value
	*N* (%)	1562	279 (18)	192 (12)	257 (16)	386 (25)	238 (16)	210 (13)	
Used FF soap/bodywash	Frequently or always	24	23	29	23	22	25	27	0.82
	Occasionally	27	27	28	26	28	27	28	
	Rarely or never	48	50	43	51	50	49	45	
Used FF skin creams	Frequently or always	21	17	23	20	22	24	24	0.004
	Occasionally	30	25	34	28	33	24	35	
	Rarely or never	49	58	42	53	45	51	41	
Used FF deodorant/antiperspirant	Frequently or always	14	14	18	15	12	9	15	0.002
	Occasionally	21	17	27	18	21	17	27	
	Rarely or never	66	69	56	67	67	74	58	
Used FF panty liners, pads, or tampons	Frequently or always	45	37	47	45	47	51	42	0.0005
	Occasionally	20	17	23	18	22	17	27	
	Rarely or never	35	46	29	37	32	32	31	
Avoid products with parabens	No	83	87	88	85	80	74	86	0.0003
	Yes	17	13	12	15	20	26	14	
Avoid products with bisphenol A	No	81	84	87	86	76	76	83	0.001
	Yes	19	16	14	14	24	24	17	
Avoid products with triclosan	No	91	90	91	95	90	90	91	0.31
	Yes	9	10	9	5	10	11	9	

Values reported are column percentages, which may not add up to 100 due to rounding. Observations with missing values were not included.

*P*-values correspond to Chi-squared test for differences in distribution across the 6 latent classes.

*FF* fragrance free.

## Discussion

In this large cohort of reproductive-aged Black individuals, we used LCA to identify subgroups of participants with distinct PCP use patterns that differed by SES. When compared to each other, the latent classes ranged in frequency of PCP use which included a class of lower overall PCP use, a class with lower overall PCP use except for higher use of nailcare products, a class with lower overall PCP use except for higher use of skin creams, a class with overall moderate PCP use, a class with higher use of makeup, haircare, and skin creams, and a class with higher overall PCP use. The starting point of this analysis was to establish groupings of participants based on PCP use and then examine the characteristics of each group. Participants who reported using PCPs more frequently were more likely to have higher SES, including higher educational attainment, higher income, and a higher likelihood of working full-time. The results of this study support and build on previous findings that Black women with higher SES were more likely to use multiple hair products [16]. This work demonstrates the importance of considering PCP exposures concurrently with other socio-demographic characteristics, lifestyle factors, and health behaviors when modeling health risks.

Prior studies have evaluated racial/ethnic differences in PCP use and provide important evidence that Black women and children are more likely to use PCPs that may contain more harmful ingredients than products used by White women and children [1, 5–7, 17, 24, 25]. For example, Black women have reported more frequent use of nailcare products compared to White women [7, 25**–**28], and use of these products has been linked to higher urinary concentrations of mono-n-butyl phthalate and mono-ethyl phthalate [29–33**]**. Some studies have also reported more frequent use of skin creams among Black women compared to White women [25, 27]; use of these products has been associated with higher urinary concentrations of parabens, phthalates, and phenols [32–37]. Parabens, phthalates, and phenols have also been linked to makeup use [32–35, 37, 38], although studies examining racial/ethnic differences in the use of makeup have reported mixed results [7, 17, 25, 27]. Black women are more likely than White women to use hair products that contain placenta (a potential source of estrogen hormones), parabens, and phthalates [3–5, 9, 13, 14]. Black women are also more likely than White women to use scented vaginal products (e.g., douches and sprays), and perfume [1, 6–8, 17, 25]. Some of these products have been found to have higher concentrations of hormonally active chemicals (e.g., parabens, phthalates, per- and polyfluoroalkyl substances (PFAS)) than products more commonly used by White women [3–5, 9]. As described in Zota et al. [9], increased use of scented vaginal douches and other fragranced intimate care products may be driven by odor discrimination--racial discrimination based on a long-standing societal myth of odors among Black women. This has been perpetuated by targeted marketing of vaginal and intimate care products towards Black women [9]. We note that only 25–30% of the SELF cohort reported regular use of vaginal products such as powder, douche, or lubricant over the previous 12 months, and use of fragrance-free panty liners, tampons, and pads was common. However, use of vaginal products was more common in this cohort when participants were younger [39]. Additional research that examines the use of both scented and fragrance-free products in conjunction with different types of discrimination (e.g., based on hairstyles or odor) is warranted.

Motivations driving PCP use decisions were not assessed in SELF, and evidence supporting SES-related differences in the frequency of PCP use, especially among Black women, is limited. More frequent use of a combination of PCPs among Black women with higher SES (e.g., use of makeup, hair products, and skin creams) may be related to lifestyle differences and/or long-standing pressures on Black women to maintain high perceived beauty standards when in professional and public settings [9, 16, 40–43]. These perceptions stem from institutionalized racism that historically embraces European beauty standards [9, 10]. For example, until 2014 the US Army banned certain hairstyles worn primarily by Black women [41]. This type of racial discrimination, reinforced by targeted marketing to Black women that promotes the use of products to lighten skin, straighten hair, or use scented vaginal products, can lead to internalized racism that influences an individual’s PCP use [9, 10, 25].

Most participants in the SELF cohort did not report avoiding products with parabens, Bisphenol-A, and triclosan. Despite data indicating that Black women are aware of toxic chemicals in PCPs, other factors such as higher cost of “clean” products, neighborhood availability, and lack of adequate labeling can preclude cleaner choices [3, 16, 24, 25, 43–45]. Despite the evidence of adverse health effects, PCPs remain poorly regulated with fragmented government oversight. Federal law currently does not require the disclosure of proprietary ingredients, such as fragrance chemicals, to consumers or regulatory agencies. However, some states, such as California, have introduced laws that remove trade secret protections and require companies to disclose chemicals in personal care and beauty products [46]. A federal bill called the Cosmetic Fragrance and Flavor Ingredient Right to Know Act of 2023–2024 [47] has been introduced to Congress and, if passed, would require companies to publicly disclose a full list of fragrance and flavor ingredients in their products on product labels and websites.

Our analysis addresses previous gaps in this literature. First, although it is important to understand racial/ethnic differences in PCP use, our study examined differences in PCP use among a cohort of Black individuals and identified related socio-economic characteristics, health behaviors, clinical characteristics, and behaviors related to product use. This information helps to build a more comprehensive understanding of how social factors may influence PCP use. This could be used to inform future research that examines how environmental factors may contribute to commonly observed health disparities and how these factors influence product availability, accessibility, and patterns of use. Second, the literature on PCPs has generally focused on single categories of products. To the best of our knowledge, this is the first study to utilize a mixture approach that captures real-world usage patterns across several categories of PCPs. Third, we used LCA to capture complex patterns of PCP use and identify distinct groups of participants with similar product use profiles. Examining the product use probabilities across groups revealed substantial differences in the use of certain products (e.g., nail products and skin cream products) that have been identified as being used more by Black women compared to White women. For example, when compared to the Lower Overall class, the Higher Nailcare class is distinguished by higher/more frequent use of nail products, and the Higher Skincare class is distinguished by higher/more frequent use of skin creams. LCA is a mixture model that accounts for correlations between PCPs such that the PCPs within classes are related but classes are independent of each other. Therefore, with control for potential confounders, these latent classes can be used as exposure variables to investigate associations between PCP use and other outcomes without the multiple-testing problems that arise when associations between individual products and other outcomes are examined. Understanding different patterns of PCP use across multiple PCP categories provides insight into whether certain patterns are associated with other risk factors for hormone-mediated health outcomes such as earlier age of menarche, breast and uterine cancer, uterine fibroids, and cardiometabolic health. Finally, no previous studies have examined how PCP patterns across different product categories differ by SES and other lifestyle factors among Black women.

Except for Gaston et al. [16], it is difficult to compare our results showing SES-related differences in PCP use among Black women to other study populations. Only a couple of studies have examined both socio-economic and racial/ethnic differences in PCP use [17, 18, 24]. However, likely due to small sample sizes, these studies did not report SES differences in PCP use by race/ethnicity or among Black women. Among studies that have evaluated socio-economic differences in PCP use and PCP-related EDC concentrations [16–18, 48, 49], there have been conflicting results in patterns of use. The findings in the current study are consistent with previous work of the same cohort that found women with higher SES were more likely to use multiple hair products [16]. Also consistent with the current study, a study of pregnant women in Ottawa, Canada reported that, compared to women with lower incomes, women with higher incomes were more likely to use more PCPs [49]. However, information on the race/ethnicity of the participants was not provided. A study of usage patterns of PCPs in California households found that women with a college education were more likely to use sunscreen, insect repellent, facial cleanser, and professional application of nail products and hair dye [18]. This study, which was majority White and <3% African American, also reported that compared to White women, African American women were more likely to have their nails professionally treated, use leave-in hair treatments, deodorant, facial cleanser, and bath gel. In several studies, compared to women with lower SES, women with higher SES had higher urinary concentrations of benzophenone-3 and triclosan [2, 48, 50, 51], chemicals often found in sunscreen, antibacterial soaps, body washes, deodorants, skin cleansers, and fluoride toothpaste (FDA.gov). In contrast to the findings in our study, a study of pregnant women (<10% non-Hispanic Black) living in Boston, Massachusetts reported that women with lower SES reported significantly higher product use, including bar soap, perfume, and nail polish [17].

This study also has several limitations. Study criteria required that participants have an intact uterus at the time of enrollment. However, participants were not queried about their gender, a socially constructed term that encompasses identity, expression, and social position with many categories beyond the binary of female and male [52, 53]. In contrast to examining the use of individual products, LCA creates manageable categorical data elements that summarize complex patterns of PCP use. However, classes can be difficult to interpret. Labels were assigned to different classes based on our observation and interpretation of the probability-based weights for class membership, and there is some subjectivity in choosing the shorthand label descriptors for different classes. Also, the categories identified with LCA are specific to the SELF-study population and may not be generalizable to other populations. Future studies examining PCP use patterns in other cohorts will help determine how PCP patterns vary across other study populations. Due to the nature of self-reported data, it is possible that PCP use in this study was misclassified. The LCA approach assigns individuals to classes based on their probability of class membership which may result in non-differential misclassification. Finally, we were unable to capture the actual products used, the chemical composition of the products, or variability in the intensity of use (e.g., heaviness of application). Although future studies may want to capture this level of information, specific product formulations change frequently likely due to changes in the availability and cost of ingredients.

## Conclusions

This study is one of the first detailed assessments of PCP usage among a large cohort of young adult Black individuals that includes multiple product categories. Participants who were more frequent users of a combination of PCPs, such as makeup, haircare, and skin creams, were more likely to have higher SES and other lifestyle and health behaviors with positive health implications. Although the latent classes are specific to this study population, the identification of socio-demographic characteristics or behaviors associated with latent classes may inform targeted and impactful exposure reduction strategies in similar populations. These findings highlight the importance of considering PCP exposures concurrently with other socio-demographic characteristics, lifestyle factors, and health behaviors when modeling health risks.

### Supplementary information

Supplementary Information

## Data Availability

Data generated or analyzed during this study can be found within the published article and its supplementary files.

## Footnotes

1 Questions relating to physical activity changed significantly between F2 and F3. Physical activity hours captured in F3 were specific to occupational activities only, whereas leisure activity hours were captured in F2. Due to this difference, we only analyzed physical activity for the subset of participants whom we had F2 data on (*N* = 1440). Physical activity at F2 was derived from four variables (total minutes of walking per week, total minutes of moderate activity per week, total minutes of vigorous activity per week, and metabolic equivalent of task* (MET) per week) (low: <1 hour/week of vigorous, 2 hours/week of moderate, and 14 hours/week of walking; low to moderate: MET score below 72; moderate: MET score at or above 72; high: 150 to 300 minutes/week of vigorous or 7 to 10 hours/week of walking; very high: at least 300 minutes/week of vigorous activity or at least 10 hours/week of moderate activity. *Metabolic equivalent of the task as defined in the U.S. Department of Health and Human Services. Physical Activity Guidelines for Americans, 2nd edition. 2018, Department of Health and Human Services;: Washington, DC: U.S.
