# Supplementary material for: Frequency of personal care product use among reproductive-aged Black individuals and associations with socio-demographic characteristics
Source: J Expo Sci Environ Epidemiol. 2024 May 29;34(4):659–69. doi: 10.1038/s41370-024-00690-x (PMC11303245; doi:10.1038/s41370-024-00690-x)
Supplement: Supplementary file 1 — Supplementary Information [file 41370_2024_690_MOESM1_ESM.docx]

**Supplemental Tables**

[**Table S1.** Distribution of socio-demographic characteristics at baseline by participant response status 2](#_Toc146712863)

[**Table S2.** Original personal care product category levels as collected in SELF questionnaire 3](#_Toc146712864)

[**Table S3.** Frequency of personal care product use category levels by product group and product type 5](#_Toc146712865)

[**Table S4.** List of personal care products considered and retained in the variable selection process. 6](#_Toc146712866)

[**Table S5.** Model fit statistics for the different number of latent classes in the variable selection algorithm 7](#_Toc146712867)

[**Figure S1.** Distribution of posterior probabilities from the LCA model by latent class. 8](#_Toc146712868)

[**Figure S2.** Strength of association between the 6 latent classes and each personal care product included in the LCA model. 9](#_Toc146712869)

# **Table S1.** Distribution of socio-demographic characteristics at baseline by participant response status

| **Characteristics** | | **Overall** | **N (%)** | |  |
| --- | --- | --- | --- | --- | --- |
|  | | **N** | **F2/F3 responders** | **Non-responders** | **P-value** |
| Age | <26 years | 378 | 345 (22) | 33 (27) | 0.6063 |
|  | 26 to <30 years | 571 | 533 (34) | 38 (31) |  |
|  | 30 to <33 years | 432 | 403 (26) | 29 (24) |  |
|  | 33 years and over | 312 | 291 (19) | 21 (17) |  |
| Marital status | Never married | 992 | 913 (58) | 79 (65) | 0.2540 |
|  | Previously married or lived as married | 236 | 220 (14) | 16 (13) |  |
|  | Currently married | 465 | 439 (28) | 26 (21) |  |
| Educational attainment | High school/GED or less | 369 | 344 (22) | 25 (21) | 0.6984 |
|  | Some college/associates/technical | 848 | 790 (50) | 58 (48) |  |
|  | Bachelors/Masters/PhD | 475 | 437 (28) | 38 (31) |  |
| Childhood: Highest | High school/GED or less | 784 | 734 (47) | 50 (41) | 0.1294 |
| educational attainment of | Some college/associates/technical | 707 | 657 (42) | 50 (41) |  |
| primary caregiver | Bachelors/Masters/PhD | 200 | 179 (11) | 21 (17) |  |
| Total annual household | <20K | 766 | 703 (45) | 63 (52) | 0.1370 |
| income | 20-50K | 628 | 593 (38) | 35 (29) |  |
|  | 50K+ | 287 | 264 (17) | 23 (19) |  |
| Current employment | Not employed | 640 | 583 (37) | 57 (47) | 0.0939 |
|  | Employed <30 hours/week | 210 | 197 (13) | 13 (11) |  |
|  | Employed 30+ hours/week | 840 | 789 (50) | 51 (42) |  |
| Body Mass Index | Less than 25 kg/m^2^ | 335 | 313 (20) | 22 (18) | 0.9035 |
|  | 25-30 kg/m^2^ | 350 | 324 (21) | 26 (21) |  |
|  | 30 to <35 kg/m^2^ | 328 | 302 (19) | 26 (21) |  |
|  | 35 kg/m^2^ and above | 680 | 633 (40) | 47 (39) |  |

Values reported in parentheses are column percentages, which may not add up to 100 due to rounding. Observations with missing values were not included.

P-values correspond to chi-square test for differences in distribution between F2/F3 responders and non-responders.

#

# **Table S2.** Original personal care product category levels as collected in SELF questionnaire

| **Product Group** | **Product Type** | **Category Levels** | | | | | | |
| --- | --- | --- | --- | --- | --- | --- | --- | --- |
|  |  | **1** | **2** | **3** | **4** | **5** | **6** | **7** |
| Makeup | Eye makeup | 2x/day or more | Once a day | 2-6x/week | 1x/week | 1-3x/month | <1x/month | Did not use |
|  | Foundation |  |  |  |  |  |  |  |
|  | Blush |  |  |  |  |  |  |  |
|  | Bronzer |  |  |  |  |  |  |  |
|  | Lipstick | 6x/day or more | 2-5x/day | 1x/day | 2-6x/week | 1x/week | 1-3x/month | Rarely or never |
|  | Lip balm |  |  |  |  |  |  |  |
|  | False eyelash | Daily | 2-6x/week | 1x/week | 1-3x/month | <1x/month | Did not use |  |
|  | Growth solution for eyelashes | Yes | No |  |  |  |  |  |
| Nailcare | Nail polish | 2x/week or more | 1/week | 1-3x/month | <1x/month | Did not use |  |  |
|  | Gel polish |  |  |  |  |  |  |  |
|  | Shellac |  |  |  |  |  |  |  |
|  | Acrylic nail ext | 12x or more in past year | 6-11x in past year | 1-5x in past year | Did not use |  |  |  |
|  | Acrylic overlays |  |  |  |  |  |  |  |
|  | Gel nail ext. |  |  |  |  |  |  |  |
|  | Gel overlays |  |  |  |  |  |  |  |
| Haircare | Hair wash (e.g., shampoo) | 2x/day or more | 1x/day | 2-6x/week | 1x/week | 1-3x/month | <1x/month | Did not use |
|  | Hair moisturizer^1^ |  |  |  |  |  |  |  |
|  | Hair styling |  |  |  |  |  |  |  |
|  | Hair coloring^2^ | >1x/month | 9-12x/year | 4-8x/year | 1-3x/year | Did not use |  |  |
|  | Hair gloss |  |  |  |  |  |  |  |
|  | Hair relaxer | 12 or more/year | 6-11x/year | 2-5x/year | 1x/year | Did not use |  |  |
| Skincare | Fade cream | 2x/week or more | 1x/week | 1-3x/month | <1x/month | Did not use |  |  |
|  | Acne cream | Daily | 2-6x/week | 1x/week | 1-3x/month | <1x/month | Did not use |  |
|  | Vaseline |  |  |  |  |  |  |  |
|  | Shea butter |  |  |  |  |  |  |  |
|  | Hand cream | 2x/day or more | 1x/day | 2-6x/week | 1x/week | 1-3x/month | <1x/month | Did not use |
|  | Face cream |  |  |  |  |  |  |  |
|  | Body cream | Daily | 2-6x/week | 1x/week | 1-3x/month | <1x/month | Did not use |  |
|  | Body oil |  |  |  |  |  |  |  |
|  | Perfume/cologne  Body spray/mist | 2x/day or more | 1x/day | 4-6x/week | 1-3x/week | 1-3x/month | Rarely or never | Did not use |
|  | Shower product | 2x/day or more | 1x/day | 2-6x/week | 1-3x/month | <1x/month |  |  |
|  | Bubble bath | 2x/week or more | 1x/week | 1-3x/month | <1x/month | Did not use |  |  |
|  | Deodorant | 2x/day or more | 1x/day | 4-6x/week | 1-3x/week | 1-3x/month | Rarely or never |  |
|  | Talc | 2x/day or more | 1x/day | 2-6x/week | 1x/week | 1-3x/month | <1x/month | Did not use |
|  | Hand sanitizer | 6x/day or more | 2-5x/day | 1x/day | 2-6x/week | 1x/week | 1-3x/month | Rarely or never |
|  | Antibacterial products (facial tissue, soap, toothpaste, toothbrush) | Daily | Frequently | Occasionally | Did not use |  |  |  |
| Vaginal products | Powder | >1x/day | Daily | 3-6x/week | 1-2x/week | 1-3x/month | <1x/month | Never |
|  | Douche |  |  |  |  |  |  |  |
|  | Lubricant | >2x/week | 1-2x/week | 1-3x/month | <1x/month | Never |  |  |

1 Hair moisturizing products include petroleum jelly, shea butter, natural plant-based oils, hair food, moisturizing creams and lotions, and conditioners.

2 Hair coloring products include henna, rinses, semi-permanent and permanent hair dyes, and hair bleach.

# **Table S3.** Frequency of personal care product use category levels by product group and product type

| **Product Group** | **Product Type ^a^** | **Category Levels** | | | |
| --- | --- | --- | --- | --- | --- |
|  |  | **Always**  **(%)** | **Often**  **(%)** | **Sometimes**  **(%)** | **Never**  **(%)** |
| Makeup | **Eye makeup** | 1x/day or more | 2-6x/week | 1x/week or less | Did not use |
|  |  | (18%) | (19%) | (35%) | (29%) |
|  | **Foundation** | 1x/day or more | 2-6x/week | 1x/week or less | Did not use |
|  |  | (10%) | (11%) | (30%) | (49%) |
|  | **Blush** | 2x/week or more | <2x/week |  | Did not use |
|  |  | (10%) | (22%) |  | (68%) |
|  | **Bronzer** | 2x/week or more | <2x/week |  | Did not use |
|  |  | (6%) | (20%) |  | (74%) |
|  | **Lipstick** | >1x/day | 1/day – 2x/week | 1x/week or less | Rarely or never |
|  |  | (13%) | (18%) | (23%) | (46%) |
|  | Lip balm | >1x/day | 1/day – 2x/week | 1x/week or less | Rarely or never |
|  |  | (50%) | (18%) | (6%) | (26%) |
|  | False eyelash | Used |  |  | Did not use |
|  |  | (42%) |  |  | (58%) |
| Nailcare | Nail polish | 1/week or more | 1-3x/month | <1x/month | Did not use |
|  |  | (19%) | (49%) | (23%) | (9%) |
|  | **Gel polish** | 1x/month or more | <1x/month |  | Did not use |
|  |  | (24%) | (17%) |  | (59%) |
|  | Shellac | 1x/month or more | <1x/month |  | Did not use |
|  |  | (18%) | (16%) |  | (66%) |
|  | Acrylic nail ext | >5x in past year | 1-5x in past year |  | Did not use |
|  |  | (14%) | (26%) |  | (60%) |
|  | **Acrylic overlays** | >5x in past year | 1-5x in past year |  | Did not use |
|  |  | (14%) | (22%) |  | (64%) |
|  | **Gel nail ext.** | >5x in past year | 1-5x in past year |  | Did not use |
|  |  | (9%) | (20%) |  | (71%) |
|  | **Gel overlays** | >5x in past year | 1-5x in past year |  | Did not use |
|  |  | (10%) | (17%) |  | (73%) |
| Haircare | Hair wash | 2x/week or more | 1x/week | 1-3x/month | <1x/month |
|  |  | (9%) | (20%) | (51%) | (20%) |
|  | **Hair moisturizer** | 1x/day or more | 1-6x/week | 3x/month or less | Did not use |
|  |  | (20%) | (34%) | (35%) | (11%) |
|  | **Hair styling** | 1x/day or more  (19%) | 1-6x/week  (31%) | 3x/month or less  (36%) | Did not use  (13%) |
|  | **Hair coloring** | 4x/year or more | 1-3x/year |  | Did not use |
|  |  | (9%) | (27%) |  | (64%) |
|  | Hair gloss | Used |  |  | Did not use |
|  |  | (10%) |  |  | (90%) |
|  | Hair relaxer | 6x/year or more | 1-5x/year |  | Did not use |
|  |  | (11%) | (35%) |  | (54%) |
| Skincare | Fade cream | Used |  |  | Did not use |
|  |  | (18%) |  |  | (82%) |
|  | Acne cream | Used |  |  | Did not use |
|  |  | (37%) |  |  | (63%) |
|  | Vaseline | 1x/day or more | 1-6x/week | 3x/month or less | Did not use |
|  |  | (29%) | (21%) | (23%) | (27%) |
|  | Shea butter | 1x/day or more | 1-6x/week | 3x/month or less | Did not use |
|  |  | (24%) | (23%) | (23%) | (29%) |
|  | **Hand cream** | 1x/day or more | <1x/day |  | Did not use |
|  |  | (58%) | (29%) |  | (13%) |
|  | **Face cream** | 1x/day or more | <1x/day |  | Did not use |
|  |  | (40%) | 453 (29%) |  | (31%) |
|  | **Body cream** | 1x/day or more | <1x/day |  | Did not use |
|  |  | (52%) | (36%) |  | (11%) |
|  | Body oil | 1x/day or more | <1x/day |  | Did not use |
|  |  | (19%) | (43%) |  | (38%) |
|  | **Perfume/cologne** | 1x/day or more | 1-6x/week |  | Rarely or never |
|  |  | (51%) | (26%) |  | (22%) |
|  | Shower product | 2x/day or more | 1x/day | <1x/day |  |
|  |  | (37%) | (49%) | (14%) |  |
|  | Bubble bath | 1x/week or more | <1x/week |  | Did not use |
|  |  | (15%) | (39%) |  | (46%) |
|  | Deodorant | 2x/day or more | 1x/day | <1x/day |  |
|  |  | (24%) | (67%) | (9%) |  |
|  | Talc | >1x/week | 1x/week or less |  | Did not use |
|  |  | (19%) | (29%) |  | (52%) |
|  | Hand sanitizer | 2x/day or more | 1x/day or 2-6x/week | 1x/week or less | Rarely or never |
|  |  | (42%) | (27%) | (19%) | (12%) |
| Vaginal products | **Powder** | 1x/month or more | <1x/month |  | Did not use |
|  |  | (24%) | (13%) |  | (63%) |
|  | **Douche** | 1x/month or more | <1x/month |  | Did not use |
|  |  | (10%) | (23%) |  | (67%) |
|  | **Lubricant** | 1x/month or more | <1x/month |  | Did not use |
|  |  | (11%) | (15%) |  | (74%) |

# **Table S4.** List of personal care products considered and retained in the variable selection process.

|  |  | Pre-screening | Final Model (pre-screened variables as starting set) | |
| --- | --- | --- | --- | --- |
| Group | Product | variables kept | starting set | variables kept |
| Makeup | Foundation | 1 | 1 | 1 |
|  | Blush |  |  | 1 |
|  | Bronzer | 1 | 1 | 1 |
|  | Eye products | 1 | 1 | 1 |
|  | False eyelash |  |  |  |
|  | Lipstick | 1 | 1 | 1 |
|  | Lip balm |  |  |  |
| Skin | Fade cream |  |  |  |
|  | Acne cream |  |  |  |
|  | Vaseline |  |  |  |
|  | Shea butter |  |  |  |
|  | Face cream | 1 | 1 | 1 |
|  | Hand cream | 1 | 1 | 1 |
|  | Body cream | 1 | 1 | 1 |
|  | Body oil |  |  |  |
|  | Perfume or body spray | 1 | 1 | 1 |
|  | Shower |  |  |  |
|  | Bubble bath |  |  |  |
|  | Deodorant |  |  |  |
|  | Talc |  |  |  |
|  | Hand sanitizer |  |  |  |
| Nails | Nail polish |  |  |  |
|  | Gel polish | 1 | 1 | 1 |
|  | Shellac |  |  |  |
|  | Acrylic nail extension |  |  |  |
|  | Gel nail extension | 1 | 1 | 1 |
|  | Acrylic overlays | 1 | 1 | 1 |
|  | Gel overlays | 1 | 1 | 1 |
| Hair | Hair wash | 1 | 1 |  |
|  | Hair cream/oil/conditioner | 1 | 1 | 1 |
|  | Hair styling products | 1 | 1 | 1 |
|  | Hair dye | 1 | 1 | 1 |
|  | Hair gloss |  |  |  |
|  | Hair relaxer in past year |  |  |  |
| Vaginal Products | Douche | 1 | 1 | 1 |
|  | Powder in genital area | 1 | 1 | 1 |
|  | Vaginal lubricant | 1 | 1 | 1 |
|  | # of variables kept |  |  | 19 |
|  | # parameters |  |  | 263 |
|  | # of latent classes |  |  | 6 |
|  | BIC |  |  | 54348.36 |
|  | -log likelihood |  |  | 26208.01 |

# **Table S5. Model fit and model diagnostic statistics** for the different number of latent classes in the variable selection algorithm, N=1,562

| **# of latent classes** | **# of parameters estimated** | **Residual df** | **Negative log likelihood** | **AIC** | **BIC** | **G^2^** | **Smallest latent class size (%)** | **Entropy** |  |  |
| --- | --- | --- | --- | --- | --- | --- | --- | --- | --- | --- |
| 2 | 87 | 1465 | 27595.97 | 55365.94 | 55831.15 | 32433.42 | 44 | 0.85 |  |  |
| 3 | 131 | 1421 | 27060.51 | 54383.02 | 55083.51 | 31362.5 | 22 | 0.86 |  |  |
| 4 | 175 | 1377 | 26645.45 | 53640.9 | 54576.68 | 30532.39 | 18 | 0.86 |  |  |
| 5 | 219 | 1333 | 26385.63 | 53209.25 | 54380.31 | 30012.74 | 14 | 0.85 |  |  |
| 6 | 263 | 1289 | 26208.01 | 52942.02 | 54348.36 | 29657.5 | 12 | 0.85 |  |  |
| 7* | 307 | 1245 | 26044.27 | 52702.54 | 54344.16 | 29330.03 | 6 | 0.86 |  |  |
| 8* | 351 | 1201 | 25893.40 | 52488.79 | 54365.69 | 29028.27 | 6 | 0.86 |  |  |
|  |  |  |  |  |  |  |  |  |  |  |

*7 and 8 latent class solutions had the lowest BIC and AIC, respectively; however, both solutions also included small latent class sizes (<10%). Therefore, we chose the next best solution of 6 clusters instead.

|  |
| --- |


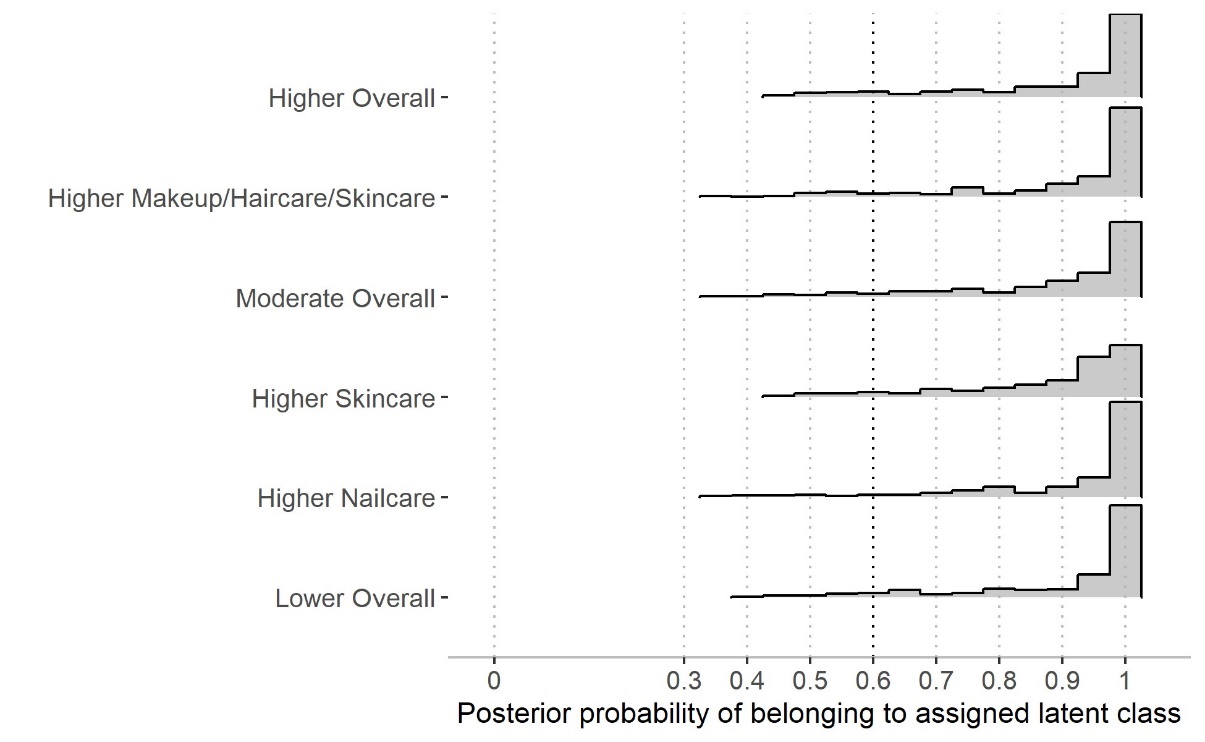


# **Figure S1.** Distribution of posterior probabilities from the LCA model by latent class. The majority of participants (> 92%) had posterior probabilities of at least p=0.60 in the class they were assigned to, 80% had probabilities over 0.80 and 69% had probabilities over 0.90, suggesting that participants were well differentiated across classes.


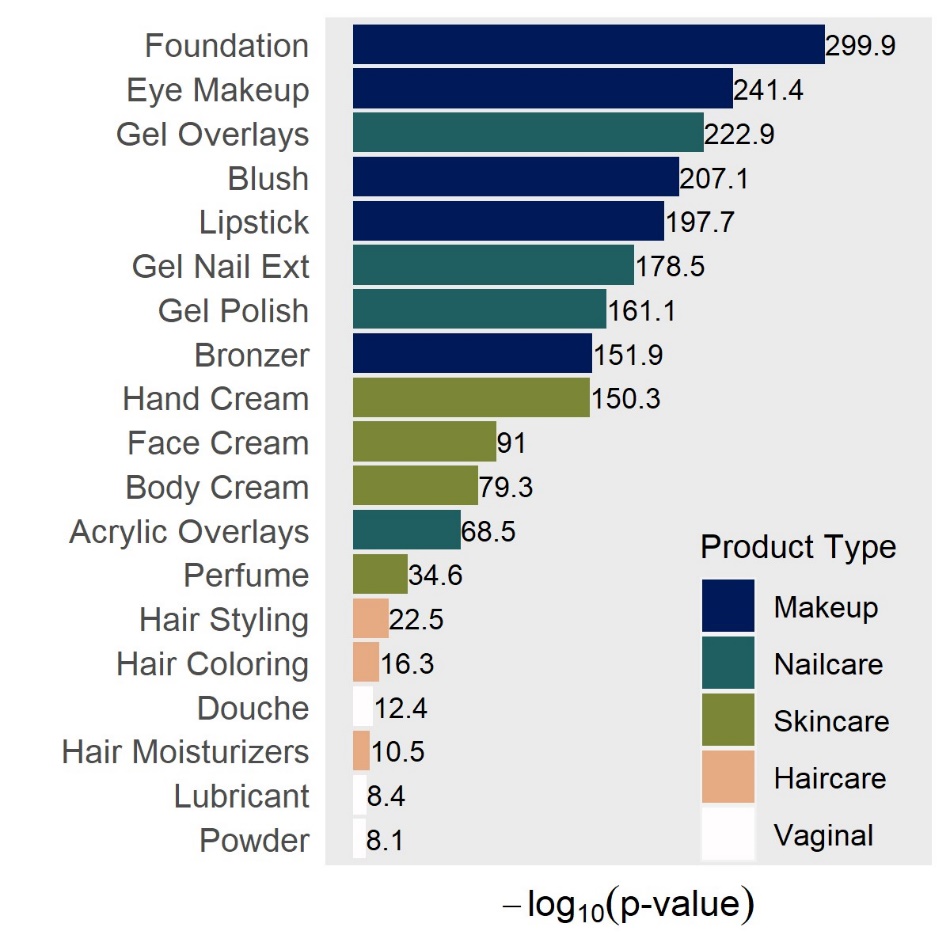


# **Figure S2.** Strength of association between the 6 latent classes and each personal care product included in the LCA model. Log worth values (or -log_10_ (p-value) from chi-squared tests, were ordered from lowest to highest, with higher log worth values indicating stronger association between the 6 latent classes and the personal care product. Makeup and nailcare products had higher log worth values than products from other categories suggesting that these products were most influential in separating the latent classes.
